# Morphological assessment of the retina in uveitis

**DOI:** 10.1186/s12348-016-0103-2

**Published:** 2016-09-09

**Authors:** Michael M. Altaweel, Sapna S. Gangaputra, Jennifer E. Thorne, James P. Dunn, Susan G. Elner, Glenn J. Jaffe, Rosa Y. Kim, P. Kumar Rao, Susan B. Reed, John H. Kempen, on behalf of the MUST Research Group

**Affiliations:** 1 Fundus Photograph Reading Center, Department of Ophthalmology and Visual Sciences, University of Wisconsin-Madison, Madison, USA; 2 Department of Ophthalmology, The Johns Hopkins University, Baltimore, MD USA; 3 Department of Epidemiology, The Johns Hopkins University, Baltimore, MD USA; 4 Kellogg Eye Center, Ann Arbor, MI USA; 5 Duke Eye Center, Durham, NC USA; 6 Retina Consultants of Houston, Houston, TX USA; 7 Department of Ophthalmology and Visual Sciences, Washington University, St. Louis, MO USA; 8 Departments of Ophthalmology and Biostatistics & Epidemiology and the Center for Clinical Epidemiology and Biostatistics, Perelman School of Medicine, The University of Pennsylvania, Philadelphia, PA USA; 9 Department of Ophthalmology and Visual Sciences, School of Medicine and Public Health, University of Wisconsin-Madison, 2870 University Ave. Suite 206, Madison, WI 53705 USA; 10 Wills Eye Hospital, Thomas Jefferson University, Philadelphia, USA

## Abstract

**Background:**

The objective of this study is to describe a system for color photograph evaluation in uveitis and report baseline morphologic findings for the Multicenter Uveitis Steroid Treatment (MUST) Trial.

Four-hundred seventy-nine eyes of 255 subjects with intermediate, posterior, and panuveitis had stereoscopic color fundus photographs obtained by certified photographers and evaluated by certified graders using standardized procedures to evaluate morphologic characteristics of uveitis. The posterior pole was evaluated for macular edema, vitreoretinal interface abnormalities, and macular pigment disturbance/atrophy; the optic disk was assessed for edema, pallor, or glaucomatous changes. The presence of neovascularization, vascular occlusion, vascular sheathing, and tractional retinal changes was determined. A random subset of 77 images was re-graded to determine the percentage agreement with the original grading on a categorical scale.

**Results:**

At baseline, 437/479 eyes had images available to grade. Fifty-three eyes were completely ungradable due to media opacity. Common features of intermediate and posterior/panuveitis were epiretinal membrane (134 eyes, 35 %), and chorioretinal lesions (140 eyes, 36 %). Macular edema was seen in 16 %. Optic nerve head and vascular abnormalities were rare. Reproducibility evaluation found exact agreement for the presence of chorioretinal lesions was 78 %, the presence and location of macular edema was 71 %, and the presence of epiretinal membrane was 71 %. Vertical cup-to-disk ratio measurement had intra-class correlation of 0.75.

**Conclusions:**

The MUST system for evaluating stereoscopic color fundus photographs describes the morphology of uveitis and its sequelae, in a standardized manner, is highly reproducible, and allows monitoring of treatment effect and safety evaluation regarding these outcomes in clinical trials.

**Electronic supplementary material:**

The online version of this article (doi:10.1186/s12348-016-0103-2) contains supplementary material, which is available to authorized users.

## Background

The Multicenter Uveitis Steroid Treatment (MUST) Trial is a randomized, partially masked, multicenter clinical trial comparing the effectiveness and safety of local therapy with the fluocinolone acetonide implant (Bausch & Lomb, Inc.) versus standardized systemic therapy with oral corticosteroids supplemented by immunosuppressive drugs when indicated for patients with severe noninfectious intermediate uveitis, posterior uveitis, or panuveitis [1].

The MUST Trial required standardized, masked evaluation of color fundus photographs of the eyes with uveitis among the patients enrolled in the study across multiple clinical centers to reliably assess the structural complications of intraocular inflammation and the morphological effects of treatment. This report describes the MUST Trial methodology for independent assessment of stereoscopic color photographs by the University of Wisconsin Fundus Photograph Reading Center (RC), provides data on the reproducibility of the evaluation procedures, and reports the baseline color fundus photograph-derived morphologic findings of the trial cohort.

## Methods

### Study participants

The study was conducted at 23 clinical sites in the USA, the United Kingdom, and Australia. The protocol and informed consent forms are compliant with the Declaration of Helsinki and were implemented under the approval of the institutional review boards governing the participating centers. Prior to the participation in the study, all clinical centers completed certification of the imaging system and certification of the participating photographer(s) through the Fundus Photograph Reading Center, Department of Ophthalmology and Visual Sciences at the University of Wisconsin-Madison. Patients were randomized on a 1:1 basis to one of the two treatment groups. Patients were imaged at baseline and study specified visits with de-identified photographs sent to the reading center for evaluation. Two-hundred fifty-five subjects were enrolled in this study; 479 eyes had uveitis at baseline. Of these, 437 eyes had gradable images. This report concentrates on the morphologic features of the evaluable eyes with uveitis at baseline.

### Image acquisition procedure

**Fig. 1:**
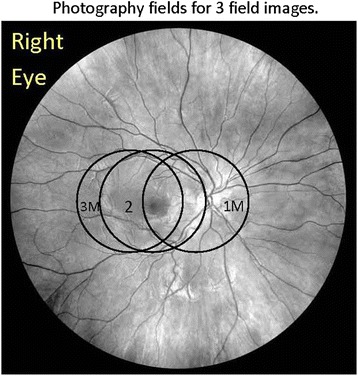
The anatomic location of the three photographic fields imaged to study the posterior pole

All patients underwent pharmacologic pupil dilation followed by three-field modified stereoscopic photography using 30° or 35° field with specified capture and export settings. The photographic fields include field 1M, where the image is centered on the temporal edge of the optic disk, field 2—centered on the macula, and field 3M—centered temporal to the center of the macula (Fig. 1). The fields have been modified from the standard Early Treatment Diabetic Retinopathy Study (ETDRS) photographic protocol [2] in order to provide additional views of the macula. At every visit, stereoscopic red reflex images were taken to document media opacities. Images were obtained in film format and sent as color slides or were obtained digitally with certified cameras and saved on a CD or DVD uncompressed and submitted to the RC according to standard procedures.

### Photo quality assessment

Film sets were viewed upon a standard light box (6500°K color temperature), using a Donaldson stereo viewer (×5). Digital images were displayed upon calibrated 20.5′′ LCD monitors and were viewed with hand-held stereo viewers (Screen-Vu Stereoscope, PS Mfg. Co., Portland, OR). Optimum image illumination, contrast, and color balance for the digital images were achieved by a standardized procedure at the RC [3] where the luminance histograms for each of the red/green/blue (RGB) color channels were analyzed and manually adjusted to enhance color contrast and standardize illumination.

Quality of both film and digital images was rated by the graders based upon the ability of the grader to view and grade different lesions of uveitis. The photographs were graded as good if image quality allowed a clear view of the retina and borderline when visibility was moderate and some features could not be graded due to quality issues such as poor stereo, poor focus, or inadequate field definition. Images were considered ungradable if there was either a very poor view or no view of the fundus.

Ungradable images were reviewed by image quality experts; on occasions where images have poor quality due to photographic technique, the clinics were contacted and photographer education and retraining were conducted. When poor image quality was due to patient-related factors such as media opacity (cataract or media haze) or confounding abnormality, the reason was documented.

### Grading procedures

Ocular disease evaluators at the reading center are trained extensively to assess and quantify lesions associated with intraocular inflammation. A certification exam is required prior to evaluation of study images, and ongoing quality control is conducted with retraining when indicated, in order to maintain a high level of quality of the data generated. Images are graded longitudinally: in grading follow-up images, the grader has access to previous images and can use the information in the decision-making for the current visit being graded. This approach allows notable changes in morphology to be identified, particularly those considered to be safety related. In grading color photographs, graders do not have access to other imaging modalities (fluorescein angiograms or optical coherence tomograms (OCT)) taken for the same subject. Evaluators are masked to treatment assignment to remove bias in morphologic assessment.

The primary objective of the MUST Trial was to compare the relative effectiveness of systemic corticosteroids plus immunosuppression versus intraocular fluocinolone acetonide implant for treatment of non-infectious intermediate, posterior, or panuveitis. The grading of color photographs was designed to permit all possible presentations of intermediate and posterior or panuveitis to be captured in a satisfactory manner and also to allow evaluation of changes in morphology over time.

The grading form was developed at the Fundus Photograph Reading Center, Madison, WI (Additional file 1), and is a modification of ETDRS diabetic retinopathy evaluation [2], with the addition of questions specific to the assessment of retinal and choroidal inflammation.

This grading procedure has been used in numerous clinical trials and has shown good to excellent reproducibility for assessment of vascular lesions and macular edema. Evaluation questions for the types of chorioretinal lesions were drafted to be descriptive and not diagnostic due to the lack of consensus on the classification of various posterior uveitides.

#### Optic nerve head abnormalities

**Fig. 2:**
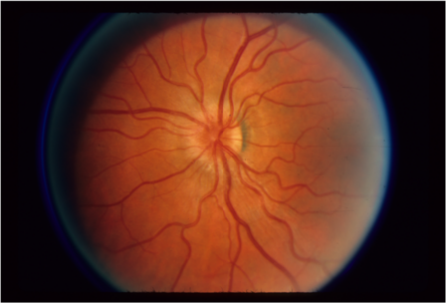
ETDRS standard photograph [2] (ETDRS example photograph C) demonstrating an optic nerve head with papillary swelling affecting 270° of the margin (from 2 o’clock to 5 o’clock)

**Fig. 3:**
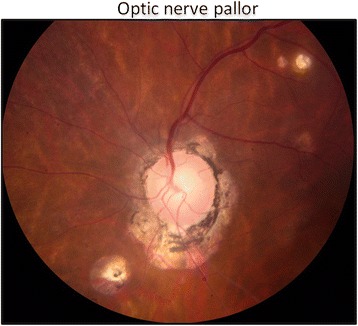
Image showing an optic nerve head with pallor. Note the constricted retinal arterioles, ghost vessels, and chorioretinal lesions

**Fig. 4:**
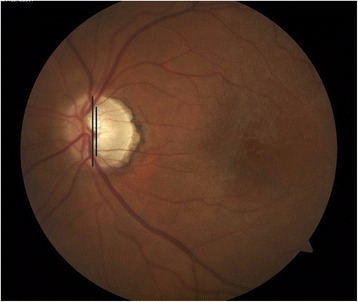
Image demonstrating the measurement of vertical cup and disk diameter in order to calculate cup-to-disk ratio

Papillary swelling may be associated with ocular inflammation. Severity of blurring of the optic disk margins is assessed by percentage involvement, as demonstrated in an ETDRS standard photograph with 270° of disk margin edema (Fig. 2). Chronic inflammation may lead to loss of optic nerve fibers and optic atrophy, characterized by optic nerve pallor (Fig. 3). Another important aspect of optic nerve morphology is the evaluation of optic nerve head cup-to-disk ratio (CDR). This may increase over time in the eyes with sustained elevation of intraocular pressure (which was expected to occur frequently in the trial). CDR is assessed by evaluating the *vertical* diameter of the optic disk margin and optic cup, using anatomic landmarks and stereoscopic photography to identify boundaries [4] (Fig. 4). Notching of the neuroretinal rim and disk hemorrhages are evaluated as additional morphologic features that may represent progressive damage of the optic nerve head due to elevated intraocular pressure.

#### Macular edema

Macular edema is a frequently noted structural complication in the eyes with uveitis and is associated with loss of visual acuity [5–7]. The assessment of macular edema requires good stereoscopic color imaging and is performed according to the procedure described for diabetic retinopathy in ETDRS Report 10 [2]. The ETDRS grid, with a size of 16 disk areas, is centered on the fovea, and the region within the grid is assessed for retinal thickening. The area involved is reported in disk areas and is determined by summing the percentage involvement within each subfield of the grid. Proximity of retinal thickening to the center of the macula and severity of thickening are assessed. For digital images, customized electronic measurement tools in Topcon IMAGEnet systems (Paramus, NJ) are used for evaluating the presence and extent of macular edema within 16 DA of the foveal center. In addition to retinal thickening, color images are graded for the presence of cystoid spaces. An important confounding factor that limits the utility of grading macular edema from color photographs in uveitis is the frequent presence of vitreoretinal interface abnormalities, such as epiretinal membrane. The appearance of retinal surface pathology makes it difficult to distinguish underlying edema and therefore precludes assessment for edema in these eyes. Grading presence and extent of macular edema in the MUST trial was more reliably accomplished with optical coherence tomography and fluorescein angiography [8].

#### Vitreoretinal interface abnormalities

**Fig. 5:**
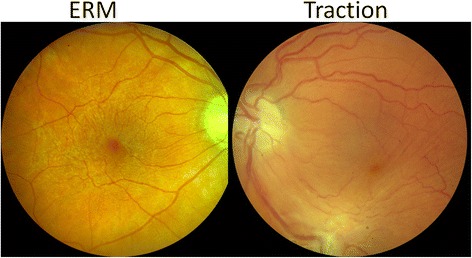
The *left panel* shows an epiretinal membrane in the macular region, and the *right panel* shows an epiretinal membrane inducing traction leading to distortion of the macula

Structural complications of the vitreoretinal interface are frequently noted sequelae of intermediate, posterior, and panuveitis. Cellophane reflex denotes a shiny appearance of the macula, is not associated with retinal traction, and is unlikely to affect visual acuity. Epiretinal membranes have more evident depth, may obscure underlying retinal detail, may be associated with retinal striae, and can be associated with tractional forces that contribute to macular edema or (in extreme cases) tractional retinal detachment (Fig. 5). Preretinal neovascularization and subsequent fibrosis are potential sequelae of chronic uveitis that can be associated with significant traction upon the retina. These complications are evaluated for their presence or absence and their effect upon the macula (see Additional file 1 for detailed grading questions).

#### Chorioretinal lesions

**Fig. 6:**
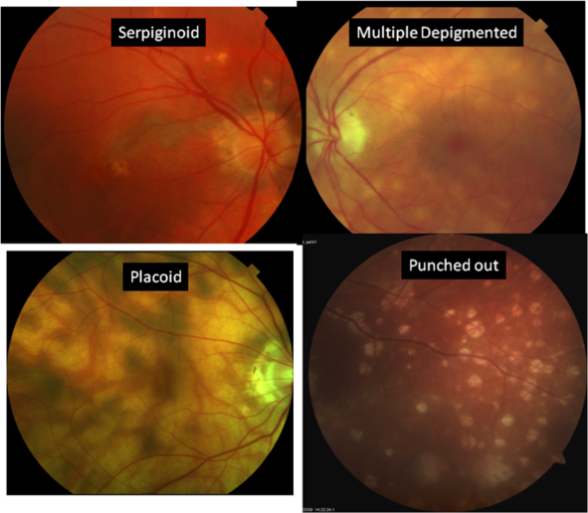
Specific subtypes of inflammatory chorioretinal lesions

An important component in the diagnosis of posterior or panuveitis and in the evaluation of severity and progression is the assessment of chorioretinal lesions. The characteristic lesions identified in the grading program are described as follows (see Fig. 6):

1. Punched-out lesions—lesions are deep, appear punched out, and may be surrounded by pigmentation. This appearance is commonly noted in multifocal choroiditis [9] and also can occur in presumed ocular histoplasmosis [10] (not studied in the MUST Trial).
2. Multiple hypopigmented round-oval lesions—multiple, shallow, cream-colored, or depigmented round or oval spots with indistinct margins, characteristically seen in birdshot chorioretinitis [11, 12].
3. Placoid lesions—flat, gray-white lesions with irregular patchy distribution present at the level of the retinal pigment epithelium (RPE), classically observed with acute posterior multifocal placoid pigment epitheliopathy (APMPPE) [13, 14].
4. Serpiginoid lesions—typically begin adjacent to the optic disk and spread peripherally in a serpentine manner with profound retinal pigment epithelial atrophy. There usually will be hyperpigmentation at the edge of the lesions [15, 16]. There may be satellite lesions.
5. Other chorioretinal lesions—lesions that do not fit well in the prior categories are termed “other” lesions. This may include scars, RPE hyperpigmentation or atrophy, or subretinal fibrosis.

**Fig. 7:**
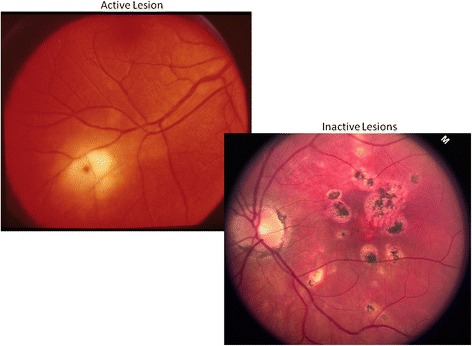
The *left panel* shows an active lesion with blurry ill-defined margins, while the *right panel* shows well-demarcated and pigmented inactive lesions

Lesion activity is graded on color photos. Active lesions generally exhibit blurred lesion margins, an appearance which contrasts sharply with inactive lesions that have sharply defined borders often with associated pigmentation (Fig. 7).

For all chorioretinal lesions, longitudinal grading is conducted for notable changes from baseline. An increase in the number of lesions, growth in area of lesions, evidence of activity, or new involvement of the fovea are identified.

#### Retinal vascular abnormalities

**Fig. 8:**
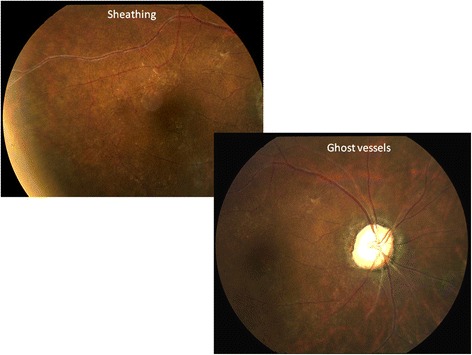
Retinal vascular changes found in the posterior segment uveitis

Retinal vascular assessment includes grading of features consistent with uveitis or subsequent complications. Retinal vessels are assessed for sheathing (sometimes a sign of activity) and sclerosis (Fig. 8). Sheathing is distinguished from sclerosis as a yellowish appearance external to the vessel wall rather than narrowing of the vessel caliber. Hemorrhages may be preretinal, subretinal, or retinal and may be associated with chorioretinal lesions or with neovascularization. Hemorrhages and neovascularization are assessed as described by ETDRS Report 10 [2]. Vascular occlusive disease may occur in the setting of uveitis; features of retinal venous or arterial occlusion are graded and identified as notable changes from baseline.

#### Reproducibility of color photograph evaluation

Quality control of MUST grading was performed by repeat grading of a random set of 77 images. For the various graded features, the calculated measures of agreement included the percent agreement (exact agreement and agreement within one step), kappa statistic (*k*), and weighted *k* statistic. Weighted *k* values were computed by assigning a weight of 1 for perfect agreement, 0.75 for one-step disagreement, and 0 for all other disagreements. Landis and Koch’s benchmarks [17] were used to evaluate simple and weighted *k* statistics in which *k* in the ranges of <0.00, 0.00–0.20, 0.21–0.40, 0.41–0.60, 0.61–0.80, 0.81–1.00, respectively, correspond to poor, slight, fair, moderate, substantial, and almost perfect agreement.

## Results

Two-hundred fifty-five subjects were enrolled at 23 clinical sites with images obtained when possible on the 479 eyes with uveitis at baseline. Of these, 437 eyes (243 subjects) had color photographs taken and were included in this analysis. Three-hundred three eyes had images acquired on film and 134 using native digital images. Among these images, 53 eyes (12 %) had poor quality which rendered all variables ungradable; thus, 88 % of eyes were imaged sufficiently well to allow assessment of the eye using this approach. The primary outcome of the MUST Trial was change in best-corrected visual acuity from baseline to 2 years. It is important to note that the ability to obtain retinal images was not an eligibility criterion for the MUST Trial in order to allow inclusion of patients with advanced and severe uveitis who would be appropriate candidates for the alternative treatments in the study. The primary reason for poor image quality was poor ocular media, due to small pupils (extensive posterior synechiae), lens opacity, and/or vitreous haze. Infrequently, image quality was poor due to photographer technique (5 %) or camera artifact.

Among the eyes assessed, optic nerve abnormalities were rare. Papillary swelling was found in 17 eyes (5 %) and optic nerve pallor in 8 eyes (2 %). Disk hemorrhages were noted in 2 eyes and notching of the rim of the optic nerve head (ONH) was found in 2 eyes. Vertical CDR was not measureable in 20 eyes (6 %), either due to poor stereoscopic view of the optic cup or confounding abnormalities on the optic nerve head. Among the eyes with measureable CDR, both the mean and median CDR was 0.3.

Assessment of the macula for macular edema was not possible in 154 of the eyes that were imaged successfully (35 %). Inability to grade was due to poor photo quality (101 eyes) or the presence of confounding abnormalities (53 eyes)—primarily the presence of an epiretinal membrane (ERM) which prevents the grader from clearly identifying whether the underlying retina is edematous on color photos. Among the 283 eyes assessed for macular edema, definite macular edema within the ETDRS grid was found in 44 eyes (16 %), with 42 eyes having edema or cysts that affected the center of the macula. ERM was gradable in 384 eyes. Cellophane reflex was noted in 48 eyes (13 %), subtle ERM in 93 eyes (24 %), and obvious ERM in 41 eyes (11 %). Traction due to epiretinal membrane was noted in 66 eyes (17 %), the most common appearance being that of striae in the retina. Distorted vessels, dragged macula due to traction, and retinal detachment were rare.

Subretinal abnormalities such as choroidal neovascularization, fibrosis, and subretinal exudation were infrequent. Chorioretinal lesions were identified in 140 of the 384 evaluable eyes (36 %). A single eye could have more than one type of chorioretinal lesion. The lesions fit the description of punched-out lesions in 32 eyes, multiple hypopigmented round-oval lesions in 59 eyes, placoid lesions in 12 eyes, and serpiginoid in 24 eyes. In addition, there were 81 eyes that had chorioretinal lesions that were not categorized into one of the lesion types described above, most commonly scars and hyperpigmented lesions. Forty-two eyes had chorioretinal lesions considered active. Retinal neovascularization and vitreous and preretinal hemorrhages were not seen in this cohort at baseline.

**Fig. 9:**
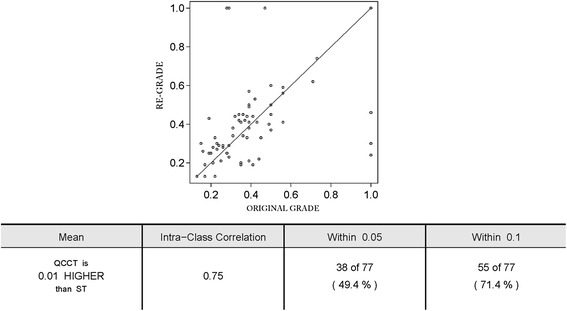
Intraclass correlation between the cup-to-disk ratio assessment in the original grade of record and the repeat quality control assessment

Table 1 presents the comparison of replicate grading (*n* = 77 eyes) with the original grading. Agreement for the presence and location of chorioretinal lesions was substantial, with 60 eyes (78 %) in exact agreement with original grade and 70 eyes (91 %) within one step (simple *k* = 0.62 (95 % CI 0.48–0.67) and weighted *k* (wk) = 0.73 (95 % CI 0.60–0.87). Macular edema agreement was assessed by evaluating the presence and location of macular edema. Fifty-five eyes (71 %) were in exact agreement (wk = 0.69, 95 % CI 0.55–0.83) and 69 eyes (90 %, wk = 0.69, 95 % CI 0.55–0.83) were within one step. Agreement regarding the height of retinal thickening at the center of the macula had exact agreement in 59 eyes (77 %; wk = 0.71, 95 % CI 0.57 = 0.85) and within one step in 68 eyes (88 %). A large proportion of the eyes randomly selected for reproducibility had epiretinal membrane. The agreement for the presence and type of epiretinal membrane was substantial, with 55 eyes (71 %) in exact agreement and 71 eyes (92 %, wk = 0.78, 95 % CI 0.67–0.88) within one step (Fig. 9).

**Table 1:** Repeat evaluation of 77 eyes as compared to the original grade of record for the assessment of key features in evaluation of color photographs for uveitis

Variable	Exact agreement, number of eyes (%)	Within one step, number of eyes (%)	Kappa (95 % CI)	wKappa (95 % CI)
Presence and extent of chorioretinal lesions^a^	60 (78 %)	70 (90.9 %)	0.62 (0.48, 0.76)	0.73 (0.60, 0.87)
Presence of retinal thickening^b^	55 (71 %)	69 (89.6 %)	0.54 (0.40, 0.69)	0.69 (0.55, 0.83)
Retinal thickening at center^c^	59 (77 %)	68 (88.3 %)	0.60 (0.46, 0.75)	0.71 (0.57, 0.85)
Presence and type of epiretinal membrane^d^	55 (71 %)	71 (92.2 %)	0.62 (0.48, 0.75)	0.78 (0.67, 0.88)

*CI* confidence interval

^a^Presence and location of chorioretinal lesions:

0—Absent

1—Questionable

2—Definite, anywhere

3—Definite, within ETDRS grid

4—Definite, at center point of the fovea

8—Cannot grade

^b^Presence of retinal thickening:

0—absent

1—questionable

2—definite, outside ETDRS grid

3—definite, within ETDRS grid

8—cannot grade

^c^Retinal thickening as center of macula:

0—absent

1—questionable

2—definite, <1× reference

3—definite, <2× reference

4—definite, >2× reference

8—cannot grade

^d^Epiretinal membrane:

0—absent

1—questionable

2—cellophane reflex

3—definite, subtle

4—definite, obvious

8—cannot grade

The most important variable from optic nerve head assessment included in the quality control analysis was the vertical CDR, an important safety outcome in a steroid treatment trial. CDR was analyzed as a continuous variable and assessed using intraclass correlation (ICC). Vertical CDR measurement had intra-class correlation of 0.75.

## Discussion

The MUST Trial compared management of intermediate, posterior, and panuveitis with fluocinolone implant versus systemic therapy with steroid and immunosuppressive medications. The spectrum of noninfectious uveitides encompassed within the trial was broad, with significant heterogeneity in morphological appearance at baseline. Fundus photographs are critical in documenting the anatomic lesions associated with uveitis. Combined with history, clinical examination, and laboratory results, retinal findings can provide adequate data to classify the uveitic entity. The Standardization of Uveitis Nomenclature working group has recommended that fundus photographs or fluorescein angiograms are required as evidence for reporting structural complications of uveitis in epidemiological trials, clinical trials, and clinical reports [18]. The Wisconsin Fundus Photograph Reading Center developed protocols which provide standardized evaluation of these anatomic features and outcomes. In the MUST Trial, clinicians are not masked to treatment assignment and hence have the potential to be biased in their assessment, and therefore RC grading of photographs is advantageous for masked objective evaluation of outcome measures. Such morphological assessment provides evidence of the structural effects of chronic uveitis, treatment response, and adverse events.

Posterior and panuveitides encompass several diseases which have different presentations and course of progression such that different management is required for each type of uveitis. For example, CMV retinitis presents with large areas of necrotizing retinitis that progresses swiftly, requiring immediate initiation of antiviral treatment, APMPPE presents with diffuse placoid lesions that are self limiting, and Birdshot chorioretinopathy presents with multiple depigmented lesions that require prompt immunosuppression. In epidemiological studies, where the focus is on the determination of type of uveitis and its natural history, the RC methodology and classification of anatomic lesions would be very useful. In therapeutic clinical trials, precise morphological characterization is also important to determine efficacy and complications of the disease. In the MUST Trial, such characterization demonstrated that the study groups were similar at baseline and serves as a method to compare the efficacy and risks of treatment between the two treatment groups.

Evaluation of the morphological features at baseline among this cohort of the eyes demonstrated a high prevalence of visually important complications of uveitis, including macular edema and vitreoretinal interface abnormalities. Assessment of chorioretinal lesions showed that although a third of the eyes had presence of lesions, involvement of the fovea was uncommon (7 eyes). CDR was within the normal range (median 0.3), and vascular lesions were rare at baseline. The high rate of ungradable photos in the MUST Trial was due to media opacity such as vitreous haze due to uveitis activity, cataract, and pupillary adhesions. The inclusion of eyes with advanced stages of severe uveitis presented a challenge to reading center grading of morphologic characteristics. In contrast, if a uveitis trial enrolls eyes that have less severe uveitis at baseline, the proportion with successful imaging and grading would be higher. In trials that have morphologic endpoints as the primary outcome, inclusion of eyes can be predicated on adequate image quality.

Among those cases that could be imaged and graded, morphological findings at baseline and through follow-up may prove useful in explaining differential response to treatment over time. If an eye has a significant epiretinal membrane and macular edema, the edema may be less likely to resolve with treatment than in eyes with macular edema but no vitreoretinal interface abnormality. Epiretinal membrane was present in 31 % of eyes at baseline. Eyes with central chorioretinal lesions would be limited in the potential to improve visual acuity despite resolution of uveitis. Such examples demonstrate the utility of accurately and thoroughly characterizing the eyes enrolled in a therapeutic trial.

The Standardization of Uveitis Nomenclature (SUN) working group [18] has advocated classifying uveitis broadly by anatomic location (anterior, intermediate, posterior, or panuveitis). Presently, there is no standardized system for the description and classification of most individual uveitic entities, resulting in ambiguity in the reporting of clinical data and poor comparability of clinical studies in the literature. The MUST Trial provides an opportunity to study the clinical characteristics and ocular complications of a large sample of eyes. Evaluation of stereoscopic retinal images at a reading center provides a uniform methodology for describing retinal lesions and changes in morphology over time that reduces the variability inherent with clinician observers in a multicenter trial.

Previous reports in uveitis literature include population-based prevalence studies [19–24] and retrospective studies on treatment modalities for uveitis. In most of these reports, the primary outcome was improvement in clinically determined inflammation as evidenced by the reduction of inflammatory cells or by using corticosteroid-sparing effect (reduction of prednisone dose to <10 mg/day) as a surrogate for control of inflammation [25–35]. Further details of fundus pathology and changes with treatment were not reported. Safety events characterized by changes in morphology were not detailed in a uniform manner. Valuable information regarding the morphological features of chronic uveitis and treatment effects could have been obtained through systematic assessment of fundus photographs. This may have been useful in indicating differential response to therapy in various subgroups as well as for monitoring for incident adverse events. In each study involving treatment with steroid implants, development of glaucoma-requiring treatment was common, with up to 40 % of the 0.59- and 2.1-mg fluocinolone dose groups combined requiring incisional surgery. These trials relied on clinician determination for the diagnosis of glaucoma. The MUST Trial obtains CDR data from photographic grading in a serial manner that provides another method of identifying morphological changes that may be attributed to glaucoma [36]. This serves as an additional safety measure for an expected side effect of intravitreal steroid administration.

An important component in the development and validation of a new methodology includes the reliability of the data collected. Review of the quality control data from the reading center shows an appropriate degree of reproducibility. The grading program required extensive grader training and certification. Standardization of the grading procedure improves the quality of data collected. Possible enhancements to the grading procedure may include integrated grading of color photos and fluorescein angiograms for the evaluation of the activity and progression of chorioretinal lesions. It also may be useful to integrate photographic and OCT findings in the evaluation of macular edema and morphology affecting the vitreoretinal interface. OCT is critical for grading macular edema as it provides a cross-sectional assessment of the macula which is not hindered by vitreoretinal interface abnormalities and moderate media opacities [37].

The strengths of the grading methodology are that it is an objective method that is reproducible and allows assessment while masked to treatment assignment. Limitations of the fundus photographic grading include the relatively small field of view captured with this protocol, compared with the overall retina. Macular edema cannot be easily graded with fundus photographs in a uveitis cohort as epiretinal membrane presence precludes the assessment of underlying retinal thickness. The solution for this in MUST was the reliance on OCT grading for determining retinal thickness and morphology associated with edema, such as cystoid spaces [37]. Patients in the MUST Trial had substantial media opacities (vitreous haze, cataract, keratitic precipitates, pupillary adhesions) that limited the number of eyes that could be adequately photographed. Despite these limitations, the MUST study provides the largest repository of morphological data obtained from a prospective uveitis clinical trial to date, allowing improved correlation of baseline and follow-up findings with other outcome measures such as visual acuity and uveitis control.

## Conclusions

In conclusion, the procedure for masked, centralized evaluation of color photographs for uveitis provides reproducible data on the characteristic lesions of intermediate and posterior uveitis suitable for use in clinical trials. The outcomes can be followed over time to evaluate treatment effects and ocular complications.

## Additional file

Additional file 1: The Detailed Grading methodolgy developed for National Eye Institute Trials. (DOCX 160 kb)
