# Supplementary material for: Morphological assessment of the retina in uveitis
Source: J Ophthalmic Inflamm Infect. 2016 Sep 9;6(1):33. doi: 10.1186/s12348-016-0103-2 (PMC5017967; doi:10.1186/s12348-016-0103-2)
Supplement: Additional file 1: — The Detailed Grading methodolgy developed for National Eye Institute Trials. (DOCX 160 kb) [file 12348_2016_103_MOESM1_ESM.docx]

**University of Wisconsin Fundus Photograph Reading Center**


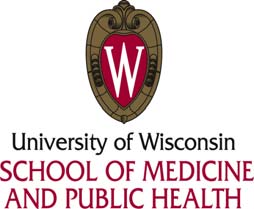


**Evaluation Procedure for Uveitis From Stereoscopic Color Fundus Images**

University of Wisconsin School of Medicine and Public Health • Department of Ophthalmology and Visual Sciences

**1. Objective**

The primary goal of this protocol is to evaluate the presence and severity of features of uveitis as documented in stereoscopic color fundus images and to describe their changes over time.

**2. Introduction**

The uveal tract is a pigmented vascular layer located between the sclera and the retina and consisting of the iris, the ciliary body, and the choroid. The uveal tract provides nutrition to the eye through the production of aqueous in the ciliary body and through provision of blood supply to the outer retina. Uveitis is a broad term referring to the inflammation of these structures.

Uveitis is classified by the structures it affects, the underlying cause, and whether it is chronic (lasting more than six weeks), or acute in nature. There are four main categories of uveitis: 1) anterior uveitis (also called iritis, the most common type), which involves the iris and the ciliary body (anterior segment of the eye); 2) intermediate uveitis, which affects the ciliary body, vitreous, and the retina (with or without mild anterior chamber involvement); 3) posterior uveitis, which involves the retina, choroid, and the optic nerve often with vitritis (inflammatory intraocular reaction in the vitreous); 4) panuveitis (diffuse uveitis), which affects the structures of the eye both in the anterior and the posterior segment with significant inflammation of the anterior chamber and vitreous.

Uveitis can result in variable clinical signs in the eye, depending on the region affected and the severity of the inflammation. Anterior segment inflammation may result in; 1) keratitic precipitates on the endothelial surface of the cornea; 2) posterior synechiae of the iris; 3) iris nodules; 4) hypopyon; and/or 5) cataract. Posterior segment changes (intermediate or posterior, or panuveitis) include: 1) vitritis, 2) chorioretinal lesions, 3) vascular sheathing, 4) macular edema, and 5) retinal detachment. The etiology of uveitis is generally in the three categories; 1) associated with systemic disease (rheumatoid arthritis, sarcoidosis), 2) associated with infections (e.g., syphilis, toxoplasmosis), or 3) idiopathic. In the United States and other developed countries the majority of intermediate uveitis, panuveitis, and about one-half of posterior uveitis cases are of idiopathic origin. Many of these are presumed to have an autoimmune etiology. Cataract and glaucoma may be major complications of uveitis.

**3. Overview**

The grading procedure described in this document is based on the Early Treatment Diabetic Retinopathy Study (ETDRS) classification of diabetic retinopathy from color fundus photographs^1^ and the Extension of ETDRS^2^. This procedure is written with the understanding that the evaluators have been trained and certified in diabetic retinopathy evaluation and in optic cup and optic disc measurement. The uveitis procedure modifies some questions and measuring tools and adds new questions. The uveitis evaluation form asks the evaluator to answer certain global questions as well as questions related to the optic disc and the macula. The images are evaluated for optic nerve abnormalities of interest such as papillary swelling, new vessels on the disc (NVD), fibrous proliferation on the disc (FPD), disc hemorrhage, pigment disturbance contiguous to the disc and optic nerve pallor. To help evaluate the presence of glaucoma, measurement for cup/disc ratio and notching of the disc questions have been added. Questions also evaluate the presence of retinal thickening (RT), cysts, hemorrhages, pigment disturbance, macular fibrosis, epiretinal membrane,

traction, hard exudates (HE) including plaque, chorioretinal lesion and type, preretinal hemorrhage (PRH), vitreous hemorrhage (VH), new vessels elsewhere (NVE), fibrous proliferation elsewhere (FPE), and vessel wall abnormalities. Information is gathered regarding the presence of other macular complications of uveitis including choroidal neovascularization (CNV), subretinal fibrosis, subretinal blood, retinal detachment, and subretinal fluid. Information concerning pigment disturbances within the grid is also gathered. Other less common abnormalities can be noted as well as *Other Abnormalities* and in longitudinal or comparative grading, as *Notable Changes* from baseline and/or previous visit.

**4. Color Grading Form**

A digital form is stored for reference and training and for evaluations when the computer program is inaccessible. *Not Applicable* is used in the computer program to differentiate between study-specific questions that are inactive in a study and questions that are being required to be graded in the uveitis study. This code gives more flexibility in an evaluation program to enable one grading form to be used for streamlined studies or very detailed studies. The procedure lists the *Not Applicable (N/A)* codes for a complete understanding of the allowable values and for the use of the evaluator in paper grading to signify the specific questions that are not required to be answered for specific studies. An asterisk (*) will be used to identify values that are gatekeeper filled. There are instances when the “Cannot Grade” value is only entered by the gatekeeper and not the evaluator. The value of 9 or series of 9s are reserved for gatekeeper values only.

For analysis, the answer *Questionable* will be equal to zero.

The Supplemental Questions are for additional questions to be added to a particular study as deemed necessary for the study by the Principal Investor.

The following describes the areas of the grading form to be completed by evaluators. The numbering used is such as to be consistent with the form.

**1. Fields Present**

In the course of multiple visit studies, 3-field, 4-wide field or 7-field photographs will be taken as part of the study protocol.

Code Description 1 3-fields 2 7-fields (incomplete) 3 7-fields (complete) 4 4-wide-angle fields (incomplete) 5 4-wide-angle fields (complete) 6 Other

**2. Camera Type**

The grader must take care to identify the camera type. FPRC evaluators use the information and examples images regarding camera types on the internal FPRC website (*FPRC Home*/*Camera types).*

**3.Can Any Fundus Views Be Evaluated?**

Due to the nature of uveitis, media opacities or a constricted pupil may be present which preclude proper photography of the fundus. This question performs as a gatekeeper by auto filling in Cannot Grade for most of the following questions in the grading form. If the retina cannot be viewed, the evaluation cannot be completed and Cannot Grade is assigned for all of the following questions with the exception of “Other Abnormalities Present” and “Notable Change from Baseline.”

Code Description

0 No

2 Yes

**4. Image Type**

The image type is important due to the different units utilized in measuring digital and film images.

Code Description

0 Film

2 Digital

**5.Papillary Swelling**

The main considerations in grading papillary swelling are severity of swelling and the extent of blurring of the disc margins. *Indeterminate* swelling is graded when intraretinal edema or blood covers the disc margin. If swelling is less severe than in FPRC ETDRS Example E **and** involves <270º of the disc margin, the grade is *3*. If swelling is as severe as that in FPRC ETDRS Example E **or** ≥270° of the disc margin, the grade is *4*.

| Code | | | | Description | | | | | | | | | | | | | | | | | | | | |
| --- | --- | --- | --- | --- | --- | --- | --- | --- | --- | --- | --- | --- | --- | --- | --- | --- | --- | --- | --- | --- | --- | --- | --- | --- |
| 0 | | | | Absent | | | | | | | | | | | | | | | | | | | | |
| 1 | | | | Questionable | | | | | | | | | | | | | | | | | | | | |
| 2 | | | | Indeterminate (due to intraretinal edema or blood) | | | | | | | | | | | | | | | | | | | | |
| 3 | | | | Definite, severity <FPRC ETDRS Ex. E **and** <270° of blurred margin | | | | | | | | | | | | | | | | | | | | |
| 4 | | | | Definite, severity ≥ FPRC ETDRS Ex. E **or** ≥ 270° of blurred margin | | | | | | | | | | | | | | | | | | | | |
| 8* | | | | CG | | | | | | | | | | | | | | | | | | | | |

**6.Optic Nerve Pallor**

Optic nerve pallor is due to loss of nerve fibers in the optic nerve, and can result from diseases of the optic nerve, retinal and/or vascular system. Disc margins are sharp. In optic nerve pallor, the disc appears white or gray in color having lost the normal pink color of the optic nerve. When the fellow eye is available, it is advisable to use the fellow eye optic disc for a comparison aid. Pay close attention to the nasal side of the disc. Normally the temporal side is paler than the nasal side.

| Code | | | | | Description | | | | | | | | | | | | | | | | | | | | | |
| --- | --- | --- | --- | --- | --- | --- | --- | --- | --- | --- | --- | --- | --- | --- | --- | --- | --- | --- | --- | --- | --- | --- | --- | --- | --- | --- |
| 0 | | | | | Absent | | | | | | | | | | | | | | | | | | | | | |
| 1 | | | | | Questionable | | | | | | | | | | | | | | | | | | | | | |
| 2 | | | | | Definite | | | | | | | | | | | | | | | | | | | | | |
| 8* | | | | | CG | | | | | | | | | | | | | | | | | | | | | |
| 9* | | | | | N/A | | | | | | | | | | | | | | | | | | | | | |

**7. Disc Hemorrhage**

Disc hemorrhages are small retinal hemorrhages that can be seen extending from within the optic nerve head to the adjacent retina. These hemorrhages typically appear blot-like when located on the disc, and more flame shaped (elongated with a feathered end) spreading from on top of the disc towards the margin of the disc. Disk hemorrhages tend to occur on the temporal side of the disc and can be found either superior temporal or inferior temporal with equal frequency. A presumed resolving disc hemorrhage will break up into blotches within the disc or at its margin and should still be considered a disc hemorrhage (even though the typical elongated shape is not present). Optic disc hemorrhages will occur in the disc or immediately adjacent to the disc. Optic disc hemorrhages will not be graded as global hemorrhages.

The grading scale is as follows:

| Code | | | | Description | | | | | | | | | | | | | | |
| --- | --- | --- | --- | --- | --- | --- | --- | --- | --- | --- | --- | --- | --- | --- | --- | --- | --- | --- |
| 0 | | | | Absent | | | | | | | | | | | | | | |
| 1 | | | | Questionable | | | | | | | | | | | | | | |
| 2 | | | | Definite | | | | | | | | | | | | | | |
| 8* | | | | CG | | | | | | | | | | | | | | |

**8.Notching of the Disc**

Thinning in one focal area of the disc can cause a “notch” to develop in the neural rim tissue (found between the cup and the edge of the disc). The normal neuroretinal rim tissue is uniformly pink in color (indicating good vascular perfusion). Because there is a round cup located in a vertically elongated oval optic disc, the width of the neural rim tissue varies by quadrant. In the normal eye, the inferior quadrant has the widest rim tissue with the superior portion second in width. The nasal tissue is slightly thinner than the superior tissue and the tissue in the temporal quadrant is the thinnest. Notching is typically seen in the inferior or the superior quadrant, as these areas are usually affected (by early glaucoma) first. Notches may range in appearance from very subtle to obvious, and more than one notch may be present. When no stereo is present, presence of notching may or may not be possible to grade.

The grading scale is as follows:

| Code | | | | Description | | | | | | | | | | | | | |
| --- | --- | --- | --- | --- | --- | --- | --- | --- | --- | --- | --- | --- | --- | --- | --- | --- | --- |
| 0 | | | | Absent | | | | | | | | | | | | | |
| 1 | | | | Questionable | | | | | | | | | | | | | |
| 2 | | | | Definite | | | | | | | | | | | | | |
| 8* | | | | CG | | | | | | | | | | | | | |

**9. Cup-to-Disc Ratio**

**Optic Cup and Optic Disc Measurements:** The optic nerve is a disc with a central depression, the cup. The cup-to disc ratio is the amount of the entire nerve head that has been cupped out. Ratios range from 0 (no cupping) to 1.0 (entire optic nerve head is cupped). Most people have some cupping, which is normal. Measurements will be of the longest meridian for the vertical measurement and shortest meridian for the horizontal measurement of the disc and of the cup. Film images will utilize the transparent strip of circles that are mentioned previously in Section 6.2 and displayed in Figure 3: Disc-Cup Circles. The circles give the measurement in thousandths (0.000) of an inch, but the measurements will be recorded in hundredths of an inch (0.00 inch). The digital images will utilize a calibrated line drawing tool for measurements. The calibration will be set in millimeters.

If either cup or disc measurement cannot be made for their respective horizontal or vertical meridian, a grade of *Cannot Grade* is given for that ratio. Papillary swelling can interfere with optic cup and/or optic disc measurements. For some studies only the vertical measurements for the optic cup and optic disc will be requested.

Cup Horizontal __.__ __ / Disc Horizontal__.__ __

= Horizontal Ratio__.__ __

Cup Vertical__.__ __ / Disc Vertical__.__ __ = Vertical Ratio __.__ __

(Cup: Film, 0.00 - 1.00 inches; Digital, 0.00 - 5.00 millimeters; both film and digital,

8.88* = CG, 9.99* = N/A)

(Disc: Film, 0.01 - 1.00 inches; Digital, 0.01 - 5.00 millimeters; both film and digital,

8.88* = CG, 9.99* = N/A)

**10. New Vessels on the Disc (NVD)**

New vessels that are clearly on the surface of the retina or disc (that is, not within the retina or disc), or further forward in the vitreous cavity, are considered to be new vessels. New vessels considered to be “on the disc” are those that both: a) originate and reside within 1 DD of the disc margin, or b) originate outside of 1 DD of the disc margin, and extend to within 1/2 DD of the disc margin when there are no other new vessels fitting the description of “a”. The NVD should have a similar density as in FPRC ETDRS Standard 10A, the NVD FPRC ETDRS standard.

The grading is based on the area involved by NVD with delineation by less than or greater or equal to 1/4 DA. This lesion is graded primarily from field 1. The grading scale is as follows:

Code Description

0 Absent 1 Questionable 2 Definite, < 1/4 DA 3 Definite, > 1/4 DA 8* CG 9* N/A

**11. Fibrous Proliferation on the Disc (FPD)**

Fibrous tissue opaque enough to be definitely seen, with or without accompanying new vessels is evaluated as fibrous proliferation. Fibrous proliferations on the surface of the disc or on the surface of the retina within 1 DD of the disc margin, or in the vitreous cavity anterior to this are considered FPD. Fibrous proliferations include fibrous strands or sheets that comprise a thickened posterior hyaloid surface, as well as completely atrophic new vessels (no visible red blood column present). Fibrous proliferation is designated on the disc (FPD) or elsewhere (FPE) using the same criteria described previously for new vessels.

In grading FPD, the area of retina covered is the main consideration, not the density or whiteness of the proliferation. This lesion is graded primarily from field 1. The grading scale is as follows:

Code Description

0 Absent 1 Questionable 3 Definite, < 1/2 DA 4 Definite, > 1/2 DA 8* CG 9* N/A

| Code | | | Description | | | | | | | | | | |
| --- | --- | --- | --- | --- | --- | --- | --- | --- | --- | --- | --- | --- | --- |
| 0 | | | Absent | | | | | | | | | | |
| 1 | | | Questionable | | | | | | | | | | |
| 2 | | | Definite | | | | | | | | | | |
| 3 | | | Atrophy contiguous to disc | | | | | | | | | | |
| 8* | | | CG | | | | | | | | | | |
| 9* | | | N/A | | | | | | | | | | |

**12. Pigment Disturbance Contiguous to Disc**

Pigment disturbance contiguous to disc includes depigmentation, atrophy, or pigment clumping (hyperpigmentation). Normal changes contiguous to the disc such as peripapillary atrophy, or temporal/myopic crescent are to be noted as code # 24 in the question “Other Abnormalities Present”, rather than being graded as present in this question. Angioid streaks, code # 20, are also to be noted in the question “Other Abnormalities Present”.

**13. Retinal Hemorrhage Presence within the Grid**

Retinal hemorrhages are patches of blood within the retina (both superficial and deep hemorrhages) and are graded based on the area of the retina covered. Hemorrhages that are anterior to the retina (i.e., pre-retinal and vitreous hemorrhages) are excluded. The grading scale for this question is as follows:

Code Description

0 Absent 1 Questionable 2 Definite, center point NOT involved, area of blood < 1/2 DA 3 Definite, center point NOT involved, area of blood > 1/2 DA 4 Definite in center subfield, center point questionably involved 5 Definite at center point, < 1/2 DA 6 Definite at center point, > 1/2 DA 8* CG

**14. Retinal Thickening Presence**

This question is a gatekeeper to the following four retinal thickness items (Retinal Thickening Proximity to Center Point, Retinal Thickening at Center of Macula, Cystoid Spaces at or near Center Point, and Retinal Thickening/Method within Grid). The grid is the standard ETDRS tic grid. Besides the stereoscopic view of the macula documented by field 2, the evaluator also examines the stereoscopic view of the parts of the macula documented in fields 1M (nasal) and 3M (temporal) and other fields for overlap of field views. (For 4W images, the evaluator uses fields 2W, 4W, and 5W.) Landmarks are identified from use of the other fields

to aid in the retinal thickening determination of location and area that is determined from field 2 (2W) (macula field).

**15. Retinal Thickening Proximity to Center Point**

The evaluator determines the proximity (in microns) of retinal thickening in relationship to the center of the macula. For film the ETDRS film distance grid is used. Digital grading utilizes the software tool available for the calibrated image. The digital images will be viewed with the preferences set to 1.80 mm equaling one disc diameter.

Retinal Thickening Proximity to Center Point = ___________μm

| Code | | | | Description | | | | | | | | | | | | | | | | | | |
| --- | --- | --- | --- | --- | --- | --- | --- | --- | --- | --- | --- | --- | --- | --- | --- | --- | --- | --- | --- | --- | --- | --- |
| 0 | | | | Absent | | | | | | | | | | | | | | | | | | |
| 1 | | | | Questionable | | | | | | | | | | | | | | | | | | |
| 2 | | | | Definite, outside grid | | | | | | | | | | | | | | | | | | |
| 3 | | | | Definite, within grid | | | | | | | | | | | | | | | | | | |
| 8* | | | | CG | | | | | | | | | | | | | | | | | | |

**16. Retinal Thickening at Center of Macula**

The presence or absence and degree of thickening at the center of the macula are recorded as *Center Thickness*. This question may be answered as *Questionable* if Retina Thickening Presence is *Questionable; Definite, outside grid*; or *Definite, inside grid*. Retinal Thickening at Center of Macula cannot be *Definite* if Retinal Thickening Presence is *Absent; Questionable*; *Definite, outside the grid*; or *Cannot Grade*. Retinal Thickening at Center of Macula can only be *Definite* if there is definite Retinal Thickening within grid.

Code Description

0 Absent

1 Questionable

2 Definite, < 1x reference

3 Definite, < 2x reference

4 Definite, > 2x reference

8* CG

**17. Cystoid Spaces At or Near Center Point**

Cystoid spaces are seen in color photographs as oval or circular areas of increased retinal transparency occurring at or near (within, 300 µm, film; 360 µm, digital) the center of the macula, usually in retinas that appear thickened. Cystoid spaces are usually round or oval with diameters 1 to 4 times that of an average major retinal vein at the disc margin. Definite cystoid spaces are defined by the visibility of at least half the circumference of a suspected cyst.

Code Description

0 Absent 1 Questionable 2 Definite 8* CG

**Direct Estimate in DA (Code 1)**

| **Subfields** | | | | | | | | **Total Area in DAs** | | | | **Allowable Values** | | | |
| --- | --- | --- | --- | --- | --- | --- | --- | --- | --- | --- | --- | --- | --- | --- | --- |
| Center + Inners + Outers (CIO) | | | | | | | |  | | | | 0 – 16.00 88.88* – CG 99.99* – N/A | | | |

**Or Subfield Percentages (Code 2)**

0 -100%, Q-Questionable, CG*-Cannot Grade, 999*-N/A

**19. Hard Exudate within the Grid**

**(If code 1-3, answer next question Hard Exudate at Center Point.)**

The grader determines whether or not the hard exudate is located within the grid. The amount of hard exudates (HE) within the grid is estimated, by comparing the amount of hard exudate within the grid to the amount to the amount of hard exudate that is in FPRC ETDRS standard

Grade hard exudate within the grid as follows:

| **Subfield** | | **Percent/Code** | | | | **Subfield** | | **Percent/Code** | |
| --- | --- | --- | --- | --- | --- | --- | --- | --- | --- |
| Center | |  | | | | N/A | | N/A | |
| IS | |  | | | | OS | |  | |
| IN | |  | | | | ON | |  | |
| II | |  | | | | OI | |  | |
| IT | |  | | | | OT | |  | |

**20. HardExudate at Center Point**

**(Answer only if previous question, “Hard Exudate in Grid”, code is 1-3.)**

The grader determines whether HE involves the center point of the macula. Available grades include the following:

Code Description

0 Absent 1 Questionable 2 Definite, not a plaque 3 Definite, plaque 8* CG 9* N/A

**21. Epiretinal Membrane**

Epiretinal membrane (also called surface wrinkling retinopathy) is the proliferation of glial tissue along the surface of the internal limiting membrane (ILM) when defects or breaks in the ILM allow cells to move into the retinal-vitreous interface. As the membrane progresses, it produces mechanical distortion of the macula. ‘Cellophane reflex’ is the fine glistening patches (or ‘wet’ appearance) overlying the macula. A grade of *Definite, subtle* references a thin whitish tissue that is beginning to obscure the vasculature. *Definite, obvious* presents a thicker whitish tissue that obscures the underlying vasculature. Contracture of the epiretinal membrane may lead to distortion of retinal vasculature, as well as macular pucker and retinal traction.

| folds. |  | | | | | | | | | | | |
| --- | --- | --- | --- | --- | --- | --- | --- | --- | --- | --- | --- | --- |
|  | Code | | Description | | | | | | | | | |
|  | 0 | | Absent | | | | | | | | | |
|  | 1  2  3  4  8 | | Questionable  Cellophane reflex  Definite, subtle  Definite, obvious  Can’t Grade | | | | | | | | | |

**22. Traction within the Grid**

As an epiretinal membrane progresses, traction at the level of the ILM creates a puckering effect. The ODE may see retinal folds radiating outward from the macula. Below (codes 2-4) are degrees of severity that can be associated with the occurrence of epiretinal membrane as well as from other causes.

Code Description

0 Absent 1 Questionable 2 Tension lines only 3 Vessel distorted 4 Dragged macula 8* CG

**23. Pigment Disturbance within the Grid**

Disturbances of the retinal pigment epithelium (RPE) sometimes lead to deposition of granules or clumps of gray or black pigment in or beneath the retina. These pigment deposits can be due to several processes including but not limited to AMD, trauma, inflammation, and

toxins or congenital. Pigment clumping due to photocoagulation is not graded as pigment disturbance. Pigment disturbance refers to either hyper- or hypo- pigmentation. Definite pigment disturbance is assessed within the grid (code2), at center point (code 3), or atrophy at center point (code 4). Pigment disturbance is measured using the standard macula grid. Atrophy is often seen in the setting of chronic cystoid macular edema. When atrophy at center point is present, code 4, it supersedes hyper- and hypo-pigmentation presence.

| Code | | Description | | | | | | | | | | |
| --- | --- | --- | --- | --- | --- | --- | --- | --- | --- | --- | --- | --- |
| 0 | | Absent | | | | | | | | | | |
| 1 | | Questionable | | | | | | | | | | |
| 2 | | Definite (hyper/hypopigmentation) | | | | | | | | | | |
| 3 | | Definite, at center point (hyper/hypopigmentation) | | | | | | | | | | |
| 4 | | Atrophy at center point (severe depigmentation) | | | | | | | | | | |
| 8* | | CG | | | | | | | | | | |
| 9* | | N/A | | | | | | | | | | |

**24.Choroidal Neovascularization**

Choroidal neovascularization (CNV) is the formation of new abnormal blood vessels in the region of the retinal pigment epithelium (RPE) (within the RPE or above it). These new vessels may cause fluid leakage and bleeding under the retina and eventual destruction of the function in the overlying retina. In some cases, CNV may appear as a gray-green tint in the retinal layer with apparent elevation of the retina or RPE in the same area. CNV is difficult to assess in color images and may require fluorescein angiography for definite assessment.

| Code | Description | | | | | |
| --- | --- | --- | --- | --- | --- | --- |
| 0 | Absent | | | | | |
| 1 | Questionable | | | | | |
| 2 | Definite, outside the grid | | | | | |
| 3 | Definite, within the grid | | | | | |
| 4 | Definite, within the center subfield | | | | | |
| 5 | Definite, at center point | | | | | |
| 8* | CG | | | | | |
| 9* | N/A | | | | | |

**25. Subretinal Fibrosis**

Subretinal fibrous tissue appears as sheets or mounds of white material under the retina. This fibrin usually proliferates in areas previously occupied by serous or hemorrhagic subretinal fluid. Fibrin appears as white material beneath the retina and may develop into scars.

| Code | Description | | | | | |
| --- | --- | --- | --- | --- | --- | --- |
| 0 | Absent | | | | | |
| 1 | Questionable | | | | | |
| 2 | Definite, outside the grid | | | | | |
| 3 | Definite, within the grid | | | | | |
| 4 | Definite, within the center subfield | | | | | |
| 5 | Definite, at center point | | | | | |
| 8* | CG | | | | | |
| 9* | N/A | | | | | |

**26. Subretinal Blood**

Subretinal blood is blood beneath the retina and so appears as a deep layer of blood, often crossing underneath retinal vessel boundaries. It may range in appearance from red to brown (if present for a long duration), to yellow/gray.

Code Description

0 Absent 1 Questionable 2 Definite, outside the grid 3 Definite, within the grid 4 Definite, within center subfield 5 Definite, at center point 8* CG 9* N/A

**27. Subretinal Fluid within the Grid**

Subretinal fluid is clear fluid that accumulates between the sensory retina and the RPE causing a blister-like elevation of the sensory retina. This separation is detected by the position of the retinal blood vessels and the partial or complete obscuring of the RPE/choroidal pattern. Obscuration of the RPE/choroidal pattern occurs when the retina is separated from the RPE, particularly when the fluid responsible for the separation is opaque, but also to some extent when it is clear. The normal retina is not perfectly transparent, and this lack of complete transparency becomes obvious when the retina is separated even slightly from the RPE. Subretinal fluid is commonly found in association with CNV.

| Code | Description | | | | | | | | |
| --- | --- | --- | --- | --- | --- | --- | --- | --- | --- |
| 0 | Absent | | | | | | | | |
| 1 | Questionable | | | | | | | | |
| 2 | Definite, within the grid | | | | | | | | |
| 3 | Definite, within center subfield | | | | | | | | |
| 4 | Definite, at center point | | | | | | | | |
| 8* | CG | | | | | | | | |
| 9* | N/A | | | | | | | | |

**28. Retinal Detachment within the Grid**

Retinal detachments are characterized by elevation of the retina from the RPE surface with accumulation of subretinal fluid. In some cases, a tractional component may be associated with retinal detachment, with striae (wrinkles or folds) on the retinal surface. Comparison with adjacent retina assists in identification of retinal detachment. (Refer to ETDRS Standard #12 for an illustration of retinal detachment). The DAs refer to the size of the detachment.

Code Description

0 Absent 1 Questionable 2 Definite, < 4 DA 3 Definite, < 8 DA 4 Definite, ≥ 8 DA 8* CG 9* N/A

**29. Retinal Detachment Outside the Grid**

The retinal fundus may be divided into four global quadrants based upon visualizing two imaginary lines drawn at right angles crossing (forming a crosshair) at the optic disc. The four global quadrants would consist of the quadrants superiorly temporal, inferiorly temporal, superiorly nasal, and inferiorly nasal to the disc.

| Code |  | | | | | | | |
| --- | --- | --- | --- | --- | --- | --- | --- | --- |
| 0 | Absent | | | | | | | |
| 1 | Questionable | | | | | | | |
| 2 | Definite, in one global quadrant | | | | | | | |
| 3 | Definite, in two global quadrants | | | | | | | |
| 4 | Definite, in three global quadrants | | | | | | | |
| 5 | Definite, in all global quadrants | | | | | | | |
| 8* | CG | | | | | | | |
| 9* | N/A | | | | | | | |

**30. Chorioretinal Lesions**

Chorioretinal lesions are assessed for presence within the eye. This is a study-specific question and thus might not be asked in all uveitis studies. This question is also a gatekeeper for the following questions: Lesion Activity, Punched Out Lesion, Multifocal Depigmented Round or Oval Lesions, Yellow Placoid Lesion, Sepiginous Lesion, and Other Chorioretinal Lesions.

Code Description

0 Absent 1 Questionable 2 Definite 3 Definite, within grid 4 Definite, at center point 8* CG 9* N/A

**31. Lesion Activity**

**(Answer only if Chorioretinal Lesion is code 2, 3, or 4.)**

Active lesions do not have distinct edges. The edges of active lesions are blurred and can be contiguous with inactive sharply edged lesions.

Code Description

0* Not active 1 Questionably active 2 Definitely active 8* CG 9* N/A

**(The chorioretinal lesion type questions that follow are answered only if Chorioretinal Lesion is code 1, 2, 3, or 4.)**

The various lesion types are descriptive of multifocal choroiditis, histoplasmosis, birdshot retinochoroidopathy, serpiginous choroidopathy, and AMPPE (acute multifocal placoid pigment epitheliopathy). A brief description of each lesion type follows.

**32. Punched Out Lesion**

**(Answer only if Chorioretinal Lesion is code 1, 2, 3, or 4.)**

Examples of Punched out Lesions are found in multifocal choroiditis and histoplasmosis: In multifocal choroiditis, lesions appear deep, punched out, have sharp margins, and are gray-white or yellow. Histoplasmosis typically has peripapillary scarring and atrophy. Peripheral punched out lesions are usually ringed either by pigment or contain pigment within the lesion.

**33. Multiple Depigmented Round or Oval Lesions**

| Code | Description | | | | | | | |
| --- | --- | --- | --- | --- | --- | --- | --- | --- |
| 0* | Absent | | | | | | | |
| 1 | Questionable | | | | | | | |
| 2 | Definite, single | | | | | | | |
| 3 | Definite, multiple | | | | | | | |
| 8* | CG | | | | | | | |
| 9* | N/A | | | | | | | |

**(Answer only if Chorioretinal Lesion is code 1, 2, 3, or 4.**)

Birdshot retinochoroidopathy lesions appear as broad, shallow, cream-colored or depigmented spots with indistinct margins. Narrowing of the retinal arterioles, retinal atrophy, optic nerve pallor, and cystoid macular edema may occur in severe chronic birdshot retinochoroidopathy. Arteriolar sheathing, optic atrophy, and choroidal neovascularization may occur.

| Code | Description | | | | | |
| --- | --- | --- | --- | --- | --- | --- |
| 0* | Absent | | | | | |
| 1 | Questionable | | | | | |
| 2 | Definite | | | | | |
| 8* | CG | | | | | |
| 9* | N/A | | | | | |

**34. Yellow Placoid Lesion(s)**

**(Answer only if Chorioretinal Lesion is code 1, 2, 3, or 4.**)

Yellow Placoid lesions are found in AMPPE, and they consist of flat, gray-white lesions present at the level of the RPE. The lesions tend to have a more irregular patchy distribution than Serpiginous lesions and are generally located closer to the fovea than Birdshot lesions.

**35. Serpiginous Lesion, Borders Near Disc (+/- Pigmented Borders)**

**(Answer only if Chorioretinal Lesion is code1, 2, 3, or 4.)**

Serpiginous choroidopathy has serpiginous lesions which typically begin concentrically around the optic disc and spread peripherally in all directions. The name draws from the serpentine crawling of the inflammatory process (the shape of the lesion) with profound retinal pigment epithelial atrophy. Serpiginous choroidopathy may spread unevenly around the macula, sparing it. Typically, retinal elevation of the lesions is minimal or nonexistent. Serpiginous choroidopathy may lead to scarring and choroidal atrophy which may result in choroidal neovascularization, with subsequent subretinal hemorrhage.

| Code | Description | | | | | |
| --- | --- | --- | --- | --- | --- | --- |
| 0* | Absent | | | | | |
| 1 | Questionable | | | | | |
| 2 | Definite | | | | | |
| 8* | CG | | | | | |
| 9* | N/A | | | | | |

**36. Other Chorioretinal Lesions**

**(Answer only if Chorioretinal Lesion is code 1, 2, 3, or 4.)**

Describe in comments the characteristics of the lesion(s). (Examples are slides # 8, Toxoplasmosis, and # 9, Active Chorioretinitis, in the FPRC Ocular Lesions and Other Grading Examples binder.)

Code Description 0 Absent 1 Questionable 2 Definite (describe in comments) 8* CG 9* N/A

**37. Retinal Hemorrhage Outside the Grid**

This does not include disc hemorrhages.

Code Description

0 Absent 1 Questionable 2 Definite, outside the grid < 1/2 DA 3 Definite, outside the grid > 1/2 DA 8* CG 9* N/A

**38. New Vessels Elsewhere (NVE)**

New vessels are defined the same as with NVD. The difference is location. New vessels that do not fit the criteria for NVD are considered NVE. New vessels elsewhere are new vessels that are more than 1DD from the disc margin and on the surface of the retina or anterior to it. If new vessels which are mostly “elsewhere” extend into the area between 1/2 and 1DD from the disc margin and no other NV are present closer to or on the disc, the grader includes all new vessels in the NVE category.

**39.Fibrous Proliferation Elsewhere (FPE)**

Fibrous proliferations (with or without new vessels) more than 1DD from the disc margin, on the surface of the retina or anterior to it, are considered FPE. If fibrous proliferations (FP) which are mostly “elsewhere” extend into the area between 1/2 and 1DD from the disc margin and no other FP are present closer to or on the disc, the grader includes all fibrous proliferations in the FPE category.

Code Description

0 Absent 1 Questionable 2 Definite, < ••• DA 3 Definite, > ••• DA 8* CG 9* N/A

**40. Preretinal Hemorrhage (PRH)**

Both boat-shaped hemorrhages with a fluid level and round, oval, or linear patches of hemorrhages just anterior to the retina or under its internal limiting membrane are included. Hemorrhage on the surface of detached retina is also considered to be PRH.

.Code Description

0 Absent 1 Questionable 2 Definite 3 Definite at center point 8* CG

**41. Vitreous Hemorrhage (VH)**

Hemorrhage further forward in the vitreous cavity than PRH, including hemorrhage on or within fibrovascular proliferations, is considered to be VH. When the haziness present could be due to hemorrhage, lens opacity, or poor focus, the grade *Questionable* is assigned. If definite hemorrhage is present, but whether it is PRH or VH is uncertain, the grade *Questionable* may not be used for both. The grader must decide which definite grade is the best choice.

Code Description

0 Absent 1 Questionable 2 Definite 8* CG

**42. Vessel Wall Abnormalities**

Venous sheathing is defined as white lines along one or both sides of the venous blood column. Arteriolar sheathing is evident when arteriolar walls are partially opaque (a ribbon of red blood can still be seen with white lines, or ‘threads’, on one or both sides of it) or completely opaque (usually a yellowish color). Sheathed vessels tend to be the same caliber as the surrounding vessel. A ghost vessel is when the whole vessel appears white, very thread­like and decreased in caliber. Sheathing and ghost vessels may both be present.

| Code | Description | | | | | |
| --- | --- | --- | --- | --- | --- | --- |
| 0 | Absent | | | | | |
| 1 | Questionable | | | | | |
| 2 | Ghost vessels present | | | | | |
| 3 | Sheathing present | | | | | |
| 4 | Ghost vessels and sheathing present | | | | | |
| 8* | CG | | | | | |
| 9* | N/A | | | | | |

**43.Other Abnormalities Present**

This section is provided to capture other abnormalities that may be of particular interest or may suggest an adverse effect of treatment. This includes changes associated with progression of glaucoma (optic nerve changes) or chorioretinitis. (Examples of code 32 are slide # 31, Central Retinal Artery Occlusion and slide #32, Branch Artery Occlusion, example of code 32 is slide #33 Central Retinal Vein Occlusion, from the FPRC Ocular Lesions and Other Grading Examples binder.)

**(If Code is a 1, 2, 3 or 4 enter additional code(s) from “Codes for Abnormalities” on Page 41.)**

**44. Notable Change from Baseline**

This section is provided to capture other changes from baseline that may be of particular interest or may suggest an adverse effect of treatment. This includes changes associated with progression of glaucoma (optic nerve changes) or chorioretinitis (increase or decrease in number of lesions).

**(If Code is a 1, 2, 3 or 4 enter additional code(s) from “Codes for Abnormalities” on Page 41.)**

|  | Code | Additional Code | Additional Code | Additional Code |
| --- | --- | --- | --- | --- |
| Absent | 0 | 99* | 99* | 99* |
| Questionable | 1 |  |  |  |
| Yes | 2 |  |  |  |
| Definite, by director review | 3 |  |  |  |
| Confirmed adverse event | 4 |  |  |  |
| CG | 8* | 88* | 88* | 88* |

**Comments**

A space is provided for describing any other features considered worthy of comment, and/or problems encountered in grading, etc. This section is left to the grader’s discretion.
